# RIG‐I promotes IFN/JAK2 expression and the endoplasmic reticulum stress response to inhibit chemoradiation resistance in nasopharyngeal carcinoma

**DOI:** 10.1002/cam4.2501

**Published:** 2019-08-28

**Authors:** Di Jing, Weibing Zhou, Lin Shen, Qian Zhang, Wang‐Ti Xie, Erdong Shen, Zhi Li, Liang‐Fang Shen, Lun‐Quan Sun

**Affiliations:** ^1^ Department of Oncology Xiangya Hospital Central South University Changsha Hunan China; ^2^ Teaching and Research Section of Surgery Xiangnan University Affiliated Hospital Chenzhou Hunan China; ^3^ Department of Oncology The First People's Hospital of YueYang Yue Yang Hunan China; ^4^ Center for Molecular Medicine Xiangya Hospital Central South University Changsha Hunan China

**Keywords:** apoptosis, endoplasmic reticulum stress, IFN/JAK2, radiotherapy and chemotherapy resistance, RIG‐I

## Abstract

RIG‐I is associated with the occurrence and development of many tumors. However, the role of RIG‐I in radiotherapy and chemotherapy in NPC has not been reported to date. In our study, RIG‐I expression was significantly reduced in chemoradiotherapy‐resistant NPC tissues and cells compared with that in therapy‐sensitive tissues and cells. RIG‐I expression increased in nonresistant NPC cells, including CNE1 and CNE2, in a dose‐dependent manner with increasing chemotherapy drug concentration or radiotherapy dose. RIG‐I overexpression promoted radiotherapy and chemotherapy sensitivity in NPC cells, leading to cellular apoptosis and increased expression of the proapoptotic factors BAX and caspase‐3. Similarly, RIG‐I knockdown in NPC cells promoted chemoradiotherapy resistance and reduced apoptosis. Analysis of microarray data indicated that the expression of IFN/JAK2 and endoplasmic reticulum (ER) stress response markers, such as JAK2, STAT1, IRF9, IFNB1, IRF3, p‐IRF3, XBP1, ATF6, IFIT2, and ISG15, was inhibited in chemoradiotherapy‐resistant cells compared with that in sensitive cells. Conversely, activation of IFN/JAK2 and ER stress response pathways in NPC cells reduced paclitaxel resistance and increased apoptosis. RIG‐I promotes IFN/JAK2 and ER stress response‐mediated apoptosis to inhibit chemoradiation resistance in nasopharyngeal carcinoma.

## Graphical abstract

RIG‐I expression was significantly reduced in chemoradiotherapy‐resistant NPC tissues and cells compared with that in therapy‐sensitive tissues and cells. RIG‐I overexpression promoted radiotherapy and chemotherapy sensitivity in NPC cells, leading to cellular apoptosis and increased expression of the proapoptotic factors BAX and caspase‐3. RIG‐I promotes IFN/JAK2 and ER stress response‐mediated apoptosis to inhibit chemoradiation resistance in nasopharyngeal carcinoma.

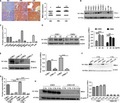

## 1 INTRODUCTION

Nasopharyngeal carcinoma (NPC) is one of the most common head and neck malignancies,^1^ with a particularly high incidence in Southern China and Southeast Asia.^2^ The 5‐year survival rate for late‐stage, nonmetastatic NPC patients is approximately 65%.^3^, ^4^, ^5^ Currently, the mainstay treatments for NPC are radiotherapy and chemotherapy.^5^ However, most patients exhibit resistance to radiotherapy and chemotherapy, resulting in treatment failure.^6^, ^7^, ^8^ Therefore, research into the mechanisms of radiotherapy and chemotherapy resistance in NPC and the identification of new targets to reduce NPC chemotherapy resistance are essential to improve treatment strategies for NPC.^9^, ^10^

Paclitaxel (Taxol, Tax) is a tetracyclic diterpenoid derived from the dried roots, leaves, and bark of the Chinese yew (*Taxus chinensis*). In 1992, paclitaxel was approved by the FDA for the treatment of ovarian cancer.^8^ As a first‐line chemotherapy option, paclitaxel can also be used in the treatment of lung cancer, melanoma,^11^, ^12^ and other cancers. Paclitaxel exhibits good efficacy in patients with NPC during the early stages of treatment, but the subsequent development of resistance is the main cause of treatment failure.^13^, ^14^, ^15^, ^16^ Therefore, more research into paclitaxel resistance mechanisms is vital.

Retinoic acid‐inducible gene I (RIG‐I) is a pattern‐recognition receptor on the cell membrane. The N‐terminus of RIG‐I contains a CARD domain that binds to MAVS on the outer mitochondrial membrane and has signal transduction capabilities. Previous studies reported that viral RNA can bind to the RIG‐I receptor, thereby activating the RIG‐I signaling pathway, as well as inflammatory and IRF3 signaling pathways, to induce IFN signaling and block viral invasion.^17^, ^18^, ^19^ Recent studies on RIG‐I in cancer have revealed that high RIG‐I expression is associated with lower survival rates in patients with pancreatic ductal carcinoma and that RIG‐I promotes cancer cell growth.^20^ We also demonstrated that EBER regulates inflammation via RIG‐I to promote NPC. However, the role of RIG‐I in NPC chemoradiation resistance has not been reported.

In the current study, we used radiotherapy‐ and chemotherapy‐resistant NPC cell lines to investigate how RIG‐I regulates chemoradiation resistance and to identify the underlying mechanisms. We found that RIG‐I regulates IFN/JAK2 and (ER) stress response‐mediated apoptosis to affect paclitaxel resistance in NPC. We propose that this mechanism may be a new target for combination drug therapy in NPC.

## 2 MATERIALS AND METHODS

### 2.1 Tissue collection

We collected nasopharyngeal epithelial tissues from 102 patients. The clinicopathological characteristics of the patients are summarized in Table 1. This study was approved by Xiangya Hospital, and all patients provided informed consent.

**Table 1:** Correlation analysis between RIG‐I expression and the clinical features of nasopharyngeal carcinoma patients

Name	Group	RIG‐I expression			*P* value
		I	II	III	
Age	<45	18	15	18	.445
	≥45	16	21	14	
Gender	male	19	24	24	.26
	female	15	12	8	
Grade	I‐II	7	6	6	.951
	III	10	9	10	
	IVa	17	21	16	
Tumor size T	T1‐T2	9	7	9	.887
	T3	12	11	10	
	T4	13	18	13	
Lymphatic metastasis N	N0‐N1	6	12	10	.126
Chemotherapeutic sensitivity	N2	12	14	16	.036^*^
	N3	16	10	6	
	CR	9	15	16	
	PR	14	13	15	
	SD	11	8	1	

### 2.2 Cell culture and treatment

Human NPC cell lines (CNE1, CNE2, HNE1, HNE2, HNE3, C666‐1, HONE1, SUNE1, HK1, 6‐10B, and 5‐8F) were maintained in our laboratory (Xiangya Hospital). All cell lines were cultured in RPMI 1640 medium (Thermo Fisher Scientific) supplemented with 10% fetal bovine serum (FBS, Thermo Fisher Scientific) at 37°C in a humidified atmosphere of 5% CO_2_. Paclitaxel‐ (CNE1‐T and CNE2‐T) and radiotherapy‐resistant (CNE1‐CR and CNE2‐CR) cell lines were generated in our laboratory.

### 2.3 Generation of paclitaxel‐resistant CNE1/T and CNE2/T and radioresistance CNE1‐CR and CNE‐CR cell sublines

The paclitaxel‐resistant NPC CNE1/T and CNE2/T cell sublines were established by exposing CNE1 and CNE2 cells to increased concentrations of paclitaxel (Cytoskeleton), as previously described.[20, 21, 22] Briefly, cells were inoculated in a 10‐mL cell culture flask and cultivated for 24 h in culture medium containing a low concentration of paclitaxel (0.1 ng/mL). Subsequently, cells were continuously cultured without paclitaxel exposure until cell growth was in the logarithmic phase. Then, cells were collected and reinoculated in a 10‐mL culture flask in culture medium containing at an elevated concentration (1.5‐ to 2‐fold of the previous dose) or at the previous concentration. This procedure was repeated until the cells exhibited stable growth and proliferation in culture medium containing 40 ng/mL paclitaxel. A period of approximately 5 months was required to establish CNE1/T and CNE2/T cell sublines. The level of drug resistance was determined using 3‐(4,5‐dimethyl‐2‐thiazolyl)‐2,5‐diphenyl‐2H‐tetrazolium bromide (MTT) assays.

To generate a radioresistant cell line, we exposed exponentially growing CNE1 and CNE2 cells to a range of IR doses (2, 4, and 6 Gy), each delivered three times at a dosage of 101.38 cGy/min. An interval of 3‐8 weeks between each IR dose allowed the surviving cells to regenerate. The entire process of IR and culture lasted approximately 1 year, and we refer to the surviving cell lines as CNE1‐CR and CNE2‐CR.^23^

### 2.4 Animals

Nude mice (4‐6 weeks old) were purchased from Hunan Jingda Laboratory Animals Co. All animals were handled according to the Guide for the Care and Use of Laboratory Animals and housed a temperature of 24 ± 1°C with 50 ± 10% humidity. All mice were randomly divided into five groups: CNE1 group, CNE1‐CR group, CNE1‐CR/RIG‐I group, CNE1‐T group, and CNE1‐T/RIG‐I group. Then, nude mice were implanted with 5 × 10^6^ NPC cells in 0.1 mL PBS to establish xenografts. Animals in the CNE1‐CR and CNE1‐CR/RIG‐I groups received 4 Gy radiation once when tumor size reached about 100 mm^3^. After 7 days, the CNE1, CNE1‐T, and CNE1‐T/RIG‐I groups were treated with paclitaxel 20 mg/kg paclitaxel every 3 days intraperitoneally (i.p.); After 7 days, the tumor size was measured every 3 days for 3 weeks. The tumor volumes were calculated using the following formula: volume = length × width × width × 0.5. Then, the mice were sacrificed and the tumors were dissected and weighted.

### 2.5 Plasmid construction and cell transfection

Human *RIG‐I* (Gene bank: NM_014314.3) cDNA was synthesized and cloned into pCDNA3.1. A pYr‐CMV‐Kan vector was used to construct a shRIG‐I lentiviral plasmid. CNE‐1 or CNE‐2 cells were infected with either OE‐RIG‐I (RIG‐I overexpression) or shRIG‐I (RIG‐I knockdown) viruses for 48 hours and then selected using 2 μg/mL puromycin for 2 weeks.^13^

### 2.6 Immunohistochemistry (IHC)

Tissues were fixed in 4% paraformaldehyde and cut into 5‐μm slices. Sections were incubated with primary antibody against RIG‐I (1:50, 25068‐1‐AP, Proteintech) for 60 minutes at 37°C. The raw value of the strongest immunostaining they could identify within the specimen on the scale 1‐3 analogous to the rules described by Rüschoff et al (1, barely visible; 2, moderate; and 3, strong).

### 2.7 RNA extraction and real‐time quantitative PCR

Total RNA was isolated using the TRIzol method following the manufacturer's protocol. RNA concentration and purity were measured on a spectrophotometer prior to cDNA synthesis. Quantitative real‐time PCR was then performed using a Real‐Time PCR Detection System (Bio‐Rad) according to the manufacturer's instructions. Values were expressed as fold changes compared with the corresponding values for the control using the 2^–ΔΔCt^ method.

### 2.8 Western blotting

Total protein was extracted from cells or tissues using RIPA lysis buffer (Auragene). Equal amounts (20 μg) of total protein were separated via 10% SDS‐PAGE, and proteins were then transferred onto a PVDF membranes. Membranes were blocked in 5% nonfat milk at room temperature for 1 hour and then incubated with primary antibodies at 4°C. Blots were subsequently incubated with secondary antibody (goat anti‐rabbit or anti‐mouse) at room temperature for 2 hour. The following primary antibodies were used: RIG‐I (1:600, 25068‐1‐AP, Proteintech); Bax (1:1000, ab32503, Abcam); caspase‐3 (1:1000, ab32087, Abcam); JAK2 (1:1000, #3230, CST); STAT1 (1:1000, #9172, CST); IRF9 (1:1000, ab51639, Abcam); IRF3 (1:1000, ab68481, Abcam); p‐IRF3 (1:500, ab76493, Abcam); XBP1 (1:1000, ab37152, Abcam); ATF6 (1:500, ab174756, Abcam); and β‐actin (1:1000, #4970, CST). Relative protein levels were quantified with respect to β‐actin.

### 2.9 Cell viability assays (MTT)

Cells were seeded onto 96‐well plates and incubated for 12 hours. Twenty microliters of MTT (Sigma) were added to each well and incubated for an additional 4 hours. Then, 200 μL of DMSO was added to each well to dissolve the crystals. Optical density was measured at 570 nm.

### 2.10 Colony‐formation assays

To test the sensitivity of cells to radiation, the cells were reseeded in six‐well plates after treatment with different doses of radiation for 48 hours and then cultured for 15 days for colony formation. Each treatment was performed in triplicate. The cell colonies were fixed in 3.7% paraformaldehyde and stained with 0.05% crystal violet solution. The dishes were photographed after staining. The cells were digested with 10% SDS, and the cell survival ratio was assessed by measuring absorbance at 570 nm.^13^

### 2.11 Apoptosis analysis by flow cytometry

Cells were harvested 24 or 48 hours after appropriate treatment. Annexin V‐FITC/propidium iodide (PI) staining was used to detect early and late apoptotic cells, as previously described.^24^, ^25^

### 2.12 Cell cycle analysis by flow cytometry

Cells plated in 12‐well plates were stained with 5 mg/mL PI (Solarbio) in PBS supplemented with RNase A for 30 minutes at room temperature and then analyzed by flow cytometry.^26^

### 2.13 Microarray analysis

CNE1/shNC and CNE1/shRIG‐I cells were treated with paclitaxel (20 μg/mL) for 24 hours. Total RNA was isolated using TRIzol reagent. GeneChip® PrimeView™ Human Gene Expression was used for gene expression analysis by Copital Biochio Corporation.

### 2.14 Statistical analysis

All data analysis was performed using GraphPad Prism version 5.0 (GraphPad Prism). The data are presented as the mean ± standard deviation (SD). The results are representative of at least three independent experiments. Analysis of differences between two groups was determined using Student's *t *test. Analysis of differences between more than two groups was performed using ANOVA. Correlation analysis between RIG‐I expression and the clinical features of nasopharyngeal carcinoma patients were determined using the kappa test. A value of *P* < .05 was defined as statistically significant.

## 3 RESULTS

### 3.1 RIG‐I expression decreased in NPC chemoradiotherapy‐resistant tissues and cells

**Figure 1:**
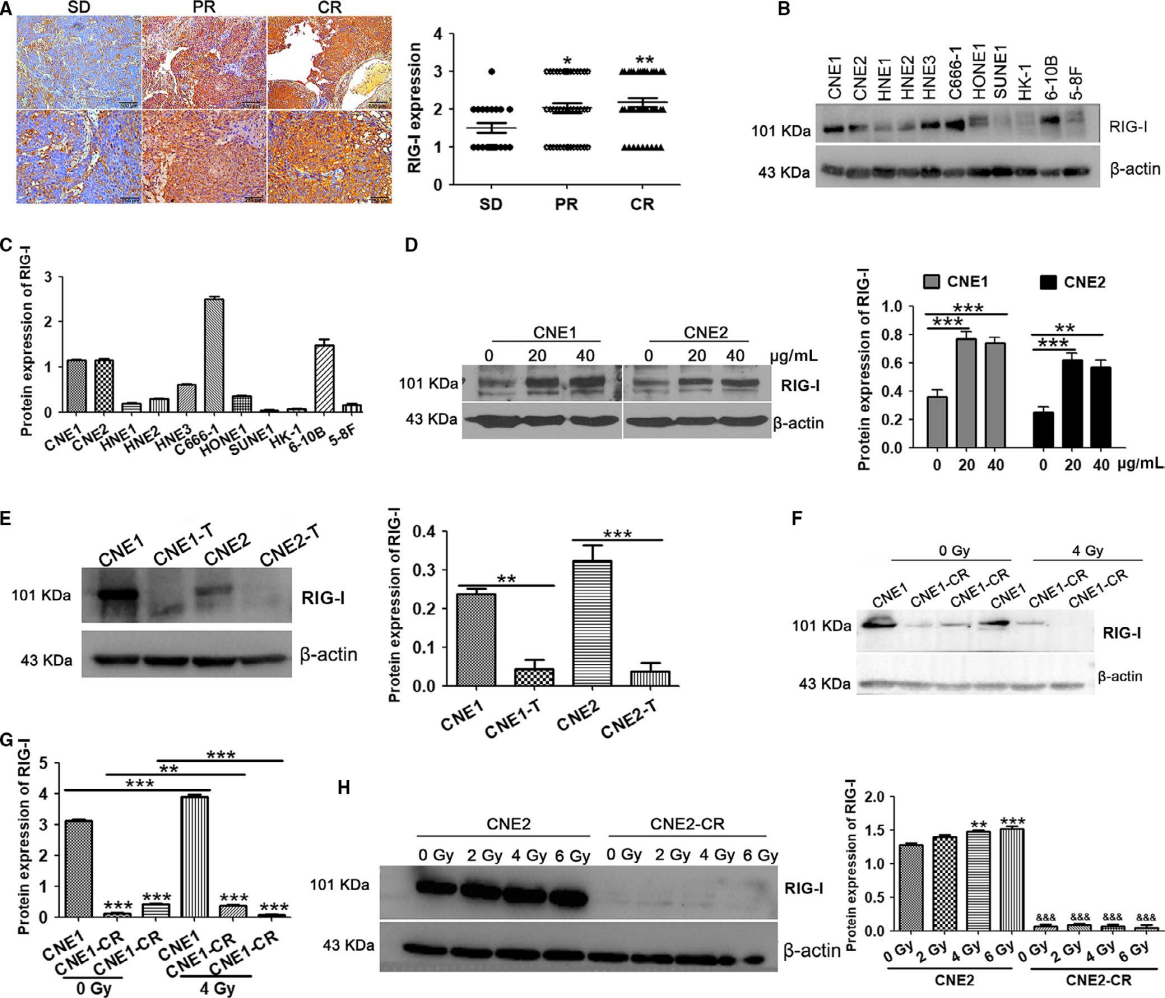
RIG‐I expression in nasopharyngeal carcinoma radiotherapy‐ and paclitaxel‐resistant tissues and cells. A, RIG‐I expression in nasopharyngeal carcinoma radiotherapy‐ and paclitaxel‐resistant and sensitive tissues was examined by IHC (SD: stable disease; PR: partial remission; CR: complete remission). B and C, RIG‐I protein expression in nasopharyngeal carcinoma cell lines (CNE1, CNE2, HNE1, HNE2, HNE3, C666‐1, HONE1, SUNE1, HK‐1, 6‐10B, and 5‐8F) detected by Western blotting. D, RIG‐I protein expression in paclitaxel‐treated CNE1 and CNE2 nasopharyngeal carcinoma cells (paclitaxel dose: 0, 20, and 40 μg/mL) at 24 h, detected by Western blotting. E, RIG‐I protein quantification in CNE1, CNE2, CNE1‐T, and CNE2‐T cells by Western blotting. F and G, RIG‐I protein expression in radiation‐treated CNE1, CNE1‐T, and CNE1‐CR nasopharyngeal carcinoma cells examined by Western blotting (radiation dose: 0, 4 Gy; duration: 48 h). H, Effects of radiation dose on RIG‐I protein expression in CNE2 and CNE2‐CR cells (radiation dose: 0 Gy, 2 Gy, 4 Gy, 6 Gy; duration: 48 h). Data represent the mean ± SEM (n = 3), *P < .05, **P < .01, ***P < .001 vs CNE1 or CNE2. *P* < .001 vs CNE2

To examine RIG‐I expression in NPC chemoradiotherapy‐sensitive or resistant tissues, we used immunohistochemistry to quantitate RIG‐I expression in NPC tissues from patients with stable disease (SD) who were chemoradiotherapy resistant and from patients with partial remission (PR) or complete remission (CR). RIG‐I expression was significantly reduced in chemoradiotherapy‐resistant tissues (Figure 1A) compared with that in sensitive tissues. No significant correlation was noted between RIG‐I expression and age, gender, grade, tumor size, or lymphatic metastasis. However, low RIG‐I expression correlated with chemoradiotherapy resistance in NPC (Table 1, *P* = .036).

Western blotting was used to assess RIG‐I expression in 11 NPC cell lines. RIG‐I was highly expressed in CNE1, CNE2, HNE3, C666‐1, and 6‐10B cell lines (Figure 1B). Next, we treated CNE1 and CNE2 cells with different doses of paclitaxel and found that RIG‐I expression increased as the paclitaxel dose increased (Figure 1B,C). In addition, we treated CNE1 and CNE2 cells with paclitaxel for different time and found that RIG‐I expression increased as the time increased (Figure S1A,B).This finding suggests that RIG‐I may play an important role in paclitaxel resistance in NPC.

Next, we examined RIG‐I expression in the paclitaxel‐resistant NPC cell lines CNE1‐T and CNE2‐T compared with that in the CNE1 and CNE2 parental cell lines. RIG‐I expression was significantly reduced in paclitaxel‐resistant CNE1‐T and CNE2‐T NPC cells compared with that in the CNE1 and CNE2 parental cells (Figure 1D). Moreover, consistent with previous studies, RIG‐I expression in the radiotherapy‐resistant CNE1‐CR and CNE2‐CR cells was significantly reduced compared with that in CNE1 and CNE2 cells (Figure 1F‐H). In addition, we treated CNE1 and CNE2 cells with radiotherapy for different time and found that RIG‐I expression increased as the time increased (Figure S1C,D).These data suggest that RIG‐I may inhibit chemoradiotherapy resistance in NPC.

### 3.2 RIG‐I regulates radiotherapy resistance in NPC cells

**Figure 2:**
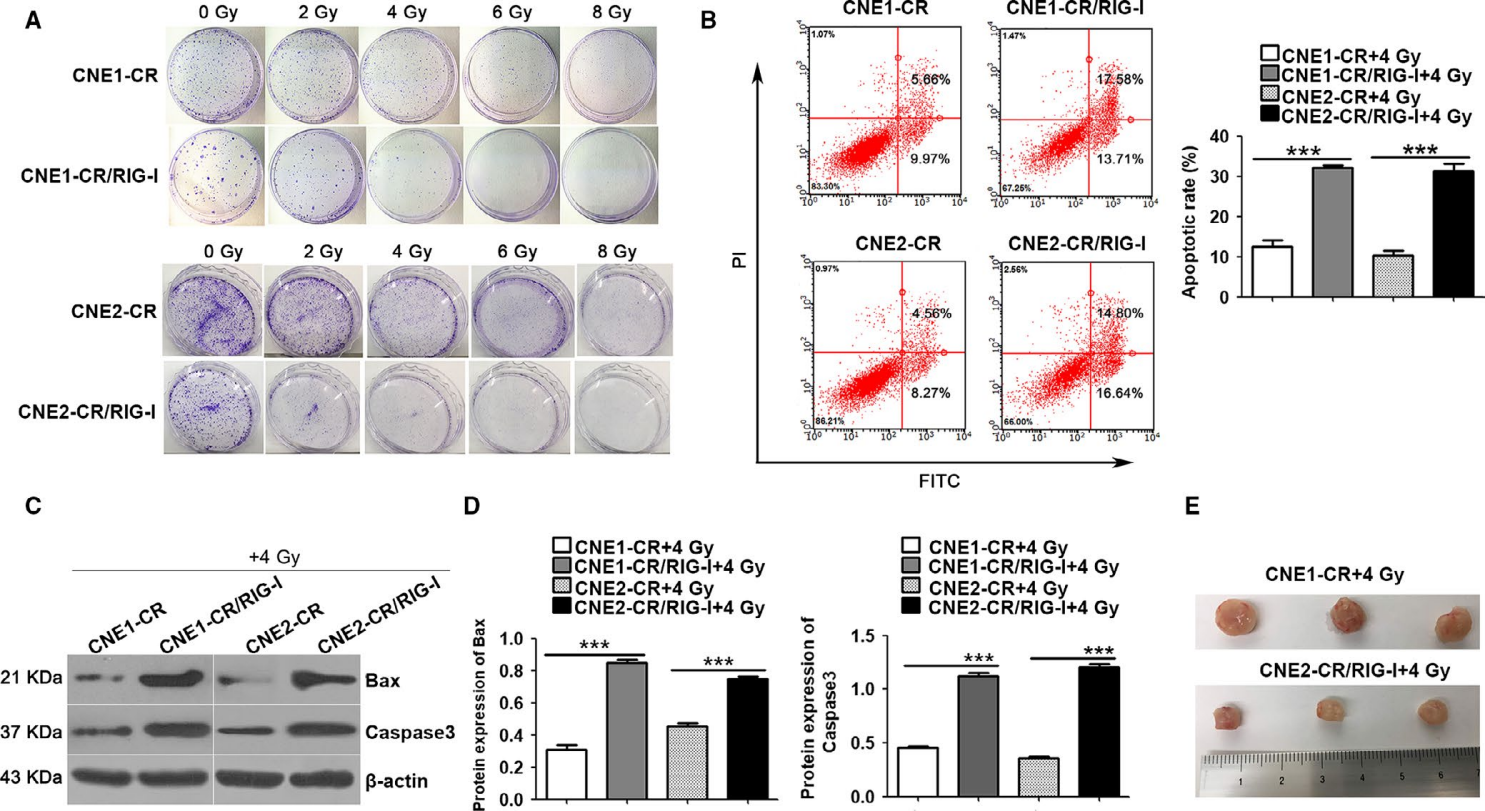
RIG‐I overexpression regulates radiotherapy resistance in nasopharyngeal carcinoma. A, Colony‐formation assay to quantify the effects of radiation dose on nasopharyngeal carcinoma cell viability (radiation dose: 0, 2, 4, 6, and 8 Gy). B, Flow cytometry analysis of the effects of radiotherapy on apoptosis in nasopharyngeal carcinoma cells after RIG‐I overexpression (radiation dose: 4 Gy, duration: 48 h). C and D, Western blot analysis of the effects of radiotherapy on proapoptotic factors (Bax and caspase‐3) in nasopharyngeal carcinoma cells after RIG‐I overexpression (radiation dose: 4 Gy, duration: 48 h). E, Nude mouse tumor growth status. Data represent the mean ± SEM (n = 3), ***P < .001 vs CNE1‐CR/ or CNE2‐CR

To examine the role of RIG‐I in radiotherapy resistance in NPC, we overexpressed RIG‐I in CNE1‐CR and CNE2‐CR cells and treated them with different doses of radiation. Colony‐formation assays were used to quantify cell proliferation. RIG‐I overexpression significantly increased the radiosensitivity of CNE1‐CR and CNE2‐CR cells (Figure 2A). We also overexpressed RIG‐I in CNE1‐CR and CNE2‐CR cells, followed by exposure to 4 Gy radiation treatment. After 48 hours, apoptosis was analyzed by flow cytometry. We found that increased RIG‐I expression promoted apoptosis in radiotherapy‐resistant NPC cells (Figure 2B). In addition, we quantified the expression of the proapoptotic factors Bax and caspase‐3 in radiotherapy‐resistant cells when RIG‐I was overexpressed under 4 Gy radiation treatment. The results indicated that Bax and caspase‐3 expression was significantly increased in RIG‐I‐overexpressing cells after radiation (Figure 2C,D). In tumor formation experiments in a xenograft model, tumors in the CNE1‐CR group were obviously larger than those in the RIG‐I‐overexpressing CNE1‐CR group at a treatment dose of 4 Gy, indicating that RIG‐I overexpression inhibited tumor growth (Figure 2E). Therefore, we hypothesize that RIG‐I increases the sensitivity of NPC radiotherapy‐resistant cells.

### 3.3 RIG‐I regulates paclitaxel resistance in NPC cells

**Figure 3:**
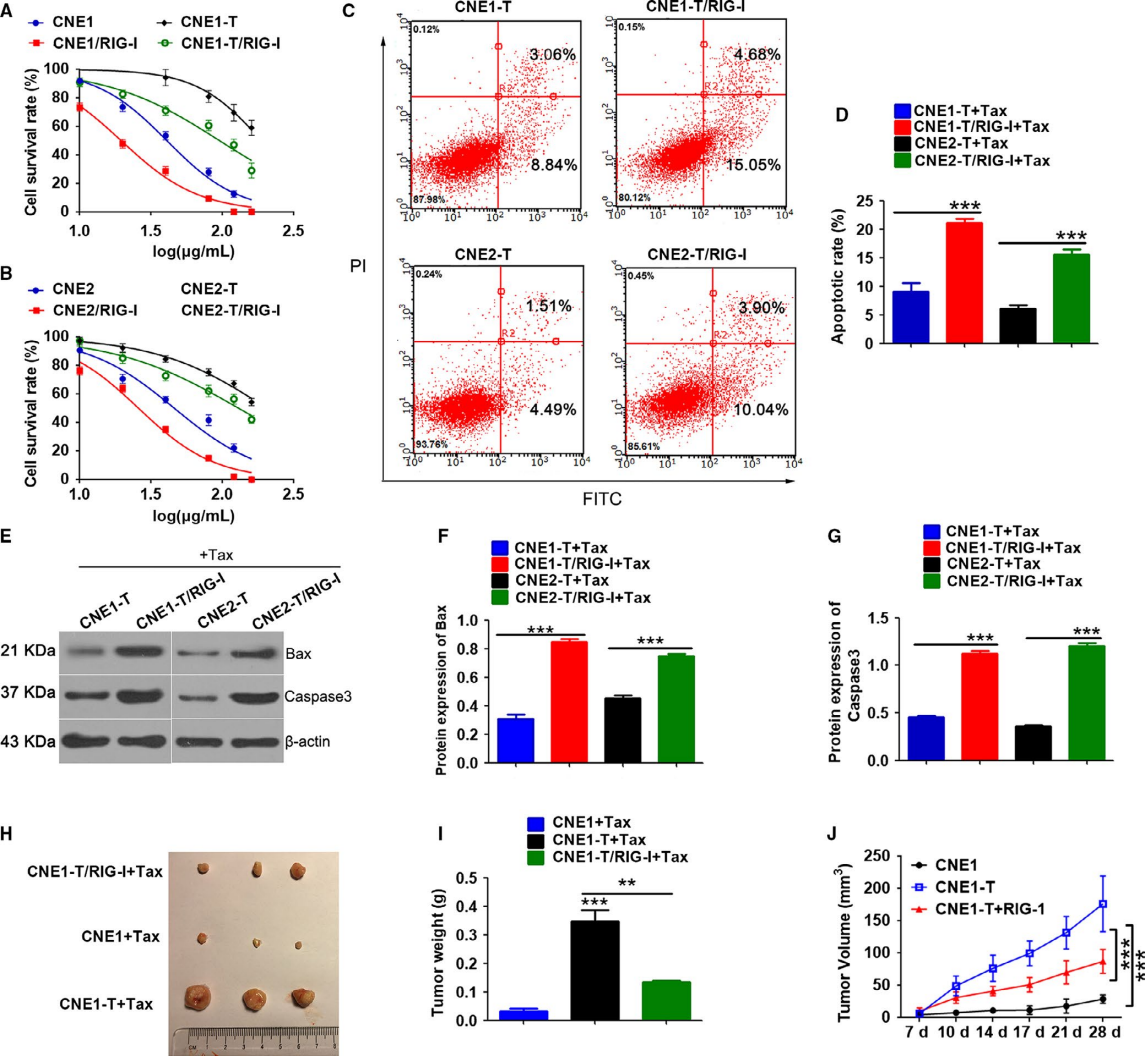
RIG‐I overexpression regulates chemotherapy resistance in nasopharyngeal carcinoma. A and B, MTT assay to quantify the effects of paclitaxel dose on viability of nasopharyngeal carcinoma cells (paclitaxel dose: 0, 10, 20, 40, 80, 120, and 160 μg/mL). C and D, Flow cytometry analysis of the effects of paclitaxel treatment on apoptosis in nasopharyngeal carcinoma cells after RIG‐I overexpression (paclitaxel dose: 20 μg/mL, duration: 24 h). E‐G, Western blot analysis of the effects of paclitaxel treatment on proapoptotic factors in nasopharyngeal carcinoma cells after RIG‐I overexpression (paclitaxel dose: 20 μg/mL, duration: 24 h). H‐J, Nude mouse tumor mass (H and I) and volume (J). (paclitaxel dose: 20 mg/kg), Data represent the mean ± SEM (n = 3), **P < .01, ***P < .001 vs CNE1‐T/ or CNE2‐T

To investigate the relationship between RIG‐I and paclitaxel resistance in NPC cells, we established paclitaxel‐resistant NPC cells that overexpress RIG‐I (CNE1‐T and CNE2‐T) (Figure S2A,B). The cells were treated with paclitaxel, and cellular apoptosis was evaluated using MTT assays, flow cytometry, and Western blotting. MTT assays revealed that RIG‐I overexpression increased the sensitivity of paclitaxel‐resistant NPC cells (Figure 3A,B). IC50 values were as follows: CNE1 vs CNE1/RIG‐I: 40.79 μg/mL vs 19.51μg/mL; CNE1‐T vs CNE1‐T\RIG‐I: 187.8 μg/mL vs 95.34 μg/mL; CNE2 vs CNE2/RIG‐I: 45.85 μg/mL vs 25.76 μg/mL; CNE2‐T vs CNE2‐T\RIG‐I: 204.4 μg/mL vs 129.3 μg/mL. Flow cytometry revealed that a greater percentage of RIG‐I‐overexpressing paclitaxel‐resistant NPC cells underwent apoptosis than was observed in the control group (Figure 3C,D). Western blotting showed that the expression of the proapoptotic factors Bax and caspase‐3 was increased in RIG‐I‐overexpressing paclitaxel‐resistant NPC cells compared with that in NPC drug‐resistant cells (Figure 3E‐G). Tumor formation experiments in nude mice revealed that RIG‐I‐overexpressing CNE‐1‐T tumors were smaller after paclitaxel treatment than those in the CNE‐1‐T control group. These results indicated that RIG‐I overexpression increases the sensitivity of paclitaxel‐resistant NPC cells and can inhibit tumor growth (Figure 3H‐J).

**Figure 4:**
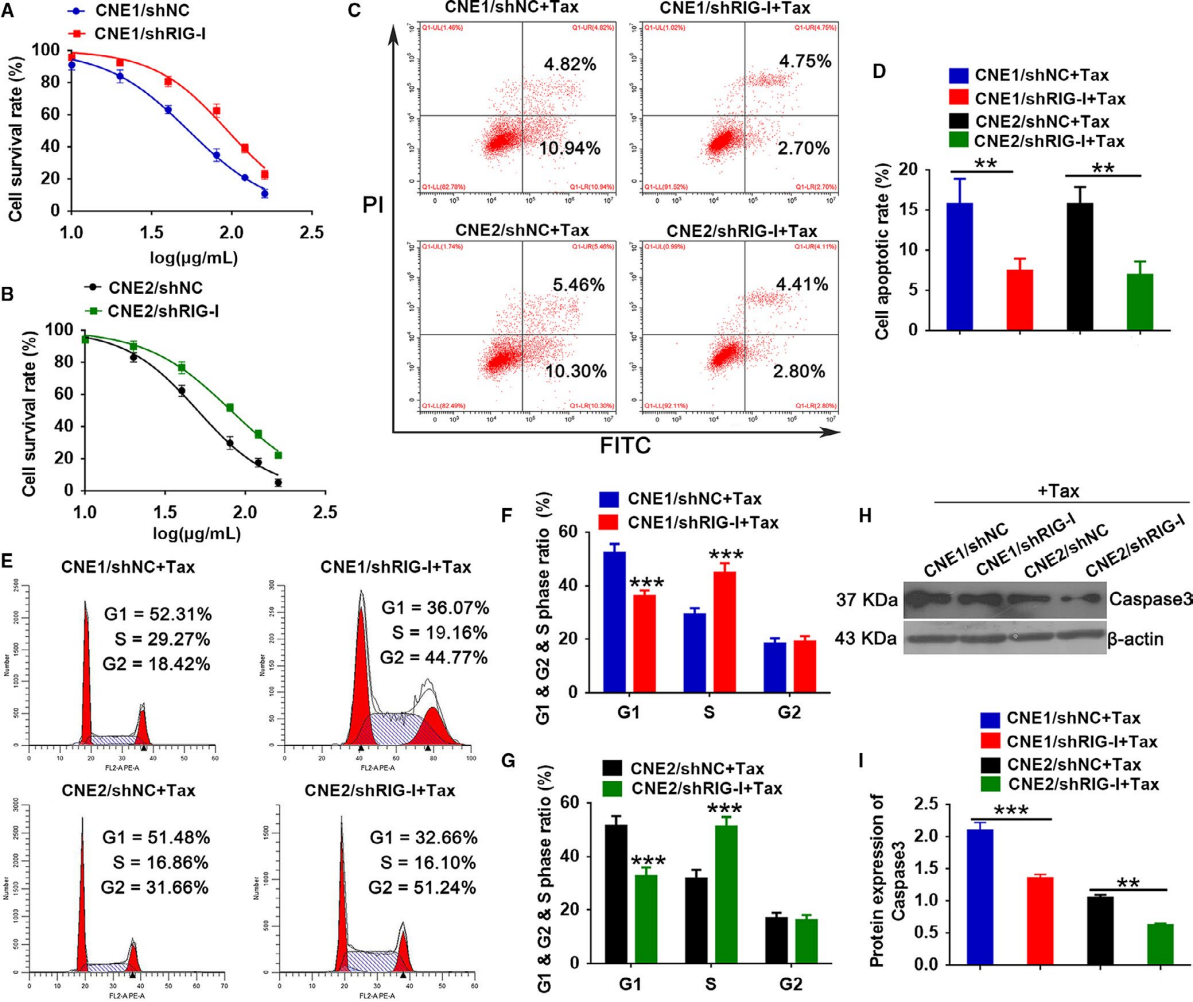
RIG‐I knockdown regulates chemotherapy resistance in nasopharyngeal carcinoma. A and B, MTT assay to quantify the effects of paclitaxel dose on viability of nasopharyngeal carcinoma cells (paclitaxel dose: 0, 10, 20, 40, 80, 120, and 160 μg/ml). C and D, Flow cytometry analysis of the effects of paclitaxel treatment on apoptosis in nasopharyngeal carcinoma cells after RIG‐I knockdown (paclitaxel dose: 20 μg/ml, duration: 24 h). E‐G, Flow cytometry analysis of the effects of paclitaxel treatment on cell cycle in nasopharyngeal carcinoma cells after RIG‐I knockdown (paclitaxel dose: 20 μg/ml, duration: 24 h). H‐I, Western blot analysis of the effects of paclitaxel treatment on caspase‐3 in nasopharyngeal carcinoma cells after RIG‐I knockdown (paclitaxel dose: 20 μg/ ml, duration: 24 h). Data represent the mean ± SEM (n = 3), **P < .01, ***P < .001 vs CNE1/ or CNE2

To further study the effects of RIG‐I on paclitaxel resistance in NPC cells, we knocked down RIG‐I in the CNE1 and CNE2 cell lines(Figure S2C,D) and evaluated the effects using MTT assays, flow cytometry analysis, and Western blotting. MTT assays revealed that RIG‐I knockdown reduced the sensitivity of NPC cells to paclitaxel (Figure 4A,B), altering IC50 values. Flow cytometry analysis revealed that apoptosis was inhibited in RIG‐I knockdown in CNE1 and CNE2 cells during paclitaxel treatment compared with that in the control group (Figure 4C,D). Cell cycle analysis revealed that CNE and CNE2 cells with RIG‐I knockdown exhibited fewer cells in G1 and more cells in the S after paclitaxel treatment than the control group. These findings indicated that reducing RIG‐I expression promotes the G1/S phase transition and cell proliferation (Figure 4E‐G), which is consistent with our previous results. Western blotting revealed that caspase‐3 expression was reduced after paclitaxel treatment in CNE1 and CNE2 cells with RIG‐I knockdown compared with that in the control group. (Figure 4H,I). In summary, these data demonstrate that RIG‐I knockdown promotes paclitaxel resistance in NPC cells.

### 3.4 RIG‐I expression inhibits JAK, IFN, and ER stress response signaling pathways

**Figure 5:**
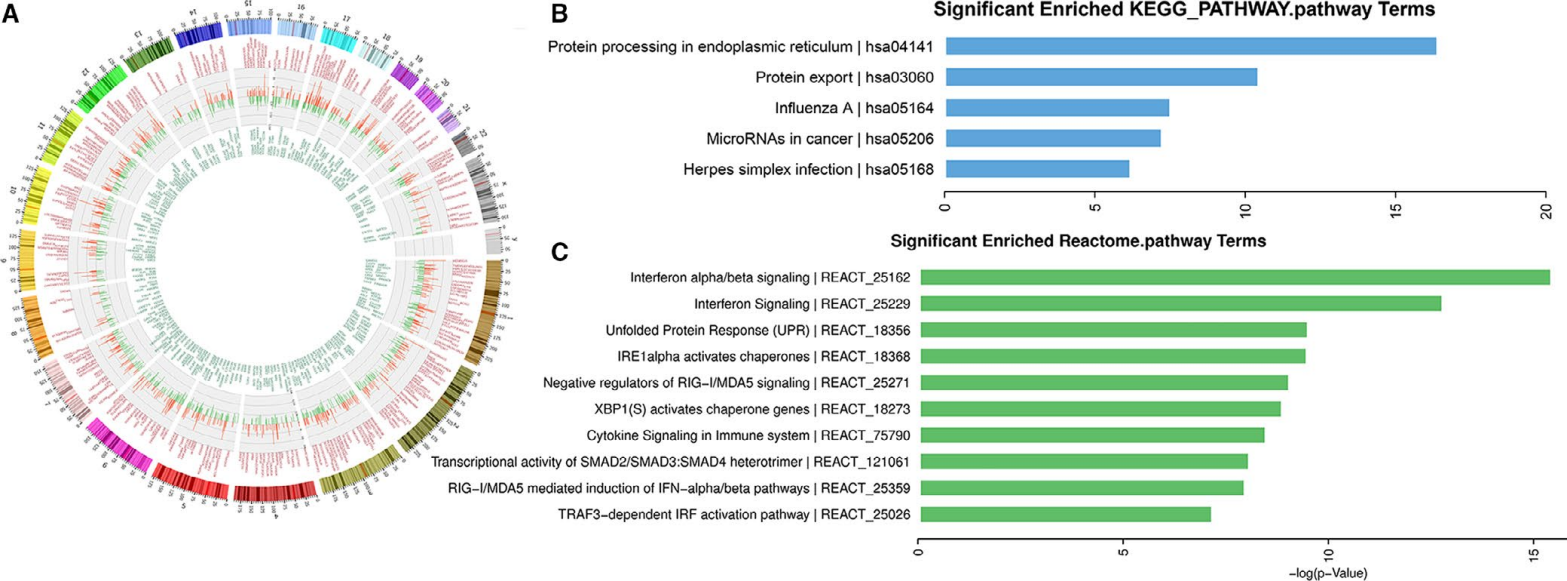
Reduced expression of JAK, IFN, and endoplasmic reticulum stress response signaling pathway factors in resistant cells. A, The differentially expressed genes in CNE1/shNC and CNE1/shRIG‐I nasopharyngeal carcinoma cells treated with paclitaxel (paclitaxel dose: 20 μg/mL, duration: 24 h). B, KEGG signaling pathway analysis of differentially expressed genes in CNE1/shNC and CNE1/shRIG‐I nasopharyngeal carcinoma cells treated with paclitaxel (paclitaxel dose: 20 μg/mL, duration: 24 h). C, Reactome signaling pathway analysis of differentially expressed genes in CNE1/shNC and CNE1/shRIG‐I nasopharyngeal carcinoma cells treated with paclitaxel (paclitaxel dose: 20 μg/mL, duration: 24 h)

**Figure 6:**
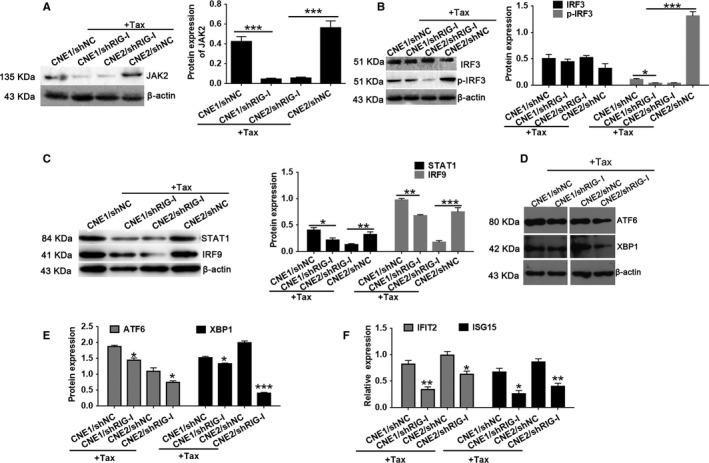
RIG‐I expression affects JAK, IFN, and endoplasmic reticulum stress response signaling pathways. A, Western blot analysis of JAK2 expression in CNE1/shNC, CNE1/shRIG‐I, CNE2/shNC, and CNE2/shRIG‐I cells after treatment with paclitaxel (20 μg/mL) for 24 h. B, Western blot analysis of IRF3 and p‐IRF3 expression in CNE1/shNC, CNE1/shRIG‐I, CNE2/shNC, and CNE2/shRIG‐I cells after treatment with paclitaxel (20 μg/mL) for 24 h. C, Western blot analysis of STAT1 and IRF9 expression in CNE1/shNC, CNE1/shRIG‐I, CNE2/shNC, and CNE2/shRIG‐I cells after treatment with paclitaxel (20 μg/mL) for 24 h. D‐E, Western blot analysis of the expression of endoplasmic reticulum stress response proteins ATF6 and XBP1 in CNE1/shNC, CNE1/shRIG‐I, CNE2/shNC, and CNE2/shRIG‐I cells after treatment with paclitaxel (20 μg/mL) for 24 h. F, qRT‐PCR analysis of the expression of IFN signaling pathway proteins ITIF2 and ISG15 in CNE1/shNC, CNE1/shRIG‐I, CNE2/shNC, and CNE2/shRIG‐I cells after treatment with paclitaxel (20 μg/mL) for 24 h. *P < .05, **P < .01, ***P < .001 vs CNE1/ shNC or CNE2/shNC

To further examine the effector mechanisms involved in RIG‐I regulation of paclitaxel resistance in NPC cells, we performed microarray experiments on paclitaxel‐treated CNE1/shNC and CNE1/shRIG‐I cells. We found that the expression of JAK, IFN, and ER stress response signaling pathway factors was reduced in these resistant cells (Figure 5A, Table 2). We also found that components of the ER stress response and IFN signaling pathways were enriched in the knockdown group (Figure 5B,C). Subsequently, we used Western blotting to quantitate the expression levels of proteins associated with the JAK2 signaling pathway (JAK2, STAT1, IRF9, IFNB1, IRF3, and p‐IRF3), as well as ER stress markers (ATF6 and Xbp1), in CNE1/shRIG‐I, CNE2/shRIG‐I, and control cells during paclitaxel treatment. In addition, qRT‐PCR assays were used to detect IFN signaling pathway‐associated factors (IFIT2 and ISG15) in CNE1/shRIG‐I, CNE2/shRIG‐I, and control cells upon paclitaxel treatment. Western blotting revealed that the expression levels of proteins associated with JAK2 signaling (JAK2, STAT1, IRF9, IFNB1, and p‐IRF3) were significantly reduced in paclitaxel‐treated CNE1/shRIG‐I and CNE2/shRIG‐I cells compared with those in the control group (Figure 6A‐C). Similar results were noted for ER stress and IFN signaling pathway‐associated factors (Figure 6D‐F). These data further validate the microarray results. We therefore hypothesize that RIG‐I regulates paclitaxel resistance in NPC cells through the JAK2/IFN and ER stress response signaling pathways.

**Table 2:** Microarray‐based detection of differentially expressed genes in CNE1/shNC and CNE1/shRIG‐I nasopharyngeal carcinoma cells treated with paclitaxel (paclitaxel dose: 20 μg/mL, duration: 24 h)

Name	shNC+Tax	shRIG‐1+Tax	Ratio	*P*
DDX58	2085.636	73.81187	28.26	0.0323
JAK2	229.6344	68.91244	3.332	0.0122
STAT1	471.7643	89.71349	5.259	0.0098
IRF9	1455.695	135.3468	10.76	0.0024
CDKN1A（P21）	3783.6	384.4544	9.842	0.0035
CDK2	331.819	429.6821	0.772	0.0093
IGFBP3	315.4426	399.7502	0.789	0.0034
GADD45A	2815.2	907.868	3.101	0.0065
IFIT2	5923.56	52.30138	113.3	0.0276
IFIT3	3539.068	71.30151	49.64	0.024
ISG15	12730.59	572.1921	22.25	0.0076
IFI44	752.0419	50.8916	14.78	0.0035
IFNB1	499.5828	50.25742	9.941	0.0054
IFI16	1816.437	192.8557	9.419	0.0265
OASL	6437.054	200.4084	32.12	0.0054
IFIT1	8929.592	352.4877	25.33	0.0198
RSAD2	736.3384	35.16483	20.94	0.0029
PPP1R15A	3032.319	207.1672		
HSPA6 /HSPA7	752.7864	53.80176	13.99	0.0067
XBP1	2166.377*	402.2313	5.386	0.0153
ATF6	273.0753**	161.6078	1.69	0.0021
JUN	934.0954	129.5761	7.209	0.0142

* *P* < .05.

** *P* < .01.

### 3.5 RIG‐I regulates paclitaxel resistance in NPC cells by regulating the JAK2/IFN and ER stress response signaling pathways

**Figure 7:**
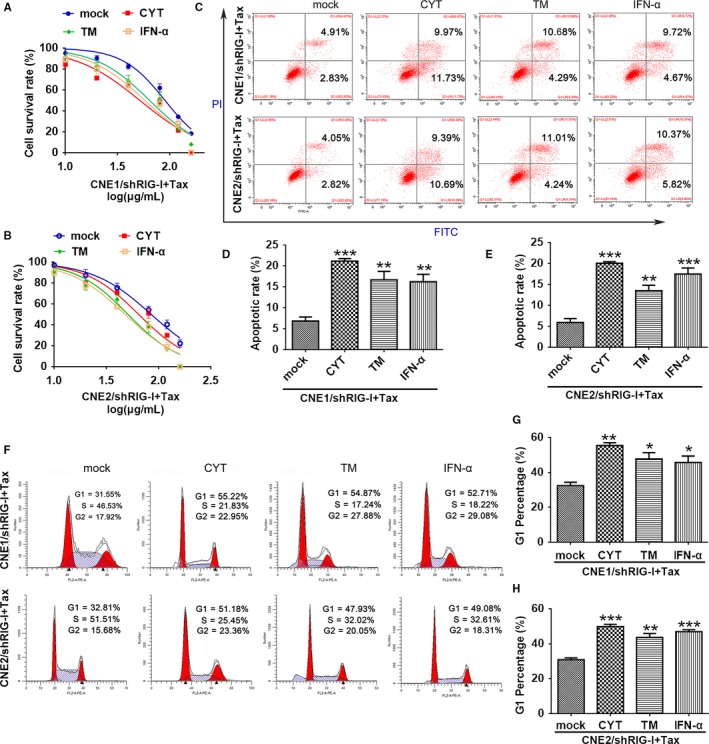
Effects of JAK2, IFN, and endoplasmic reticulum stress response signaling pathways on paclitaxel resistance in nasopharyngeal carcinoma. A and B, CNE1/shRIG‐I (A) and CNE2/shRIG‐I (B) cells were treated with CYT, TM, and IFN and different concentrations of paclitaxel for 24 h. MTT assays were used to quantify cell proliferation (paclitaxel dose: 0, 10, 20, 40, 80, 120, and 160 μg/mL; JAK activator: CYT; endoplasmic reticulum stress activator: TM; IFN signaling pathway activator: IFN‐α). C‐E, CNE1/shRIG‐I (A) and CNE2/shRIG‐I (B) cells were cotreated with CYT, TM, IFN, and paclitaxel (20 μg/mL) for 24 h. Flow cytometry was used to quantify apoptosis. F‐H, CNE1/shRIG‐I (A) and CNE2/shRIG‐I (B) cells were cotreated with CYT, TM, IFN, and paclitaxel (20 μg/mL) for 24 h. Flow cytometry was used to identify cell cycle stages. Data represent the mean ± SEM (n = 3), *P < .05, **P < .01, ***P < .001 vs CNE1/shRIG‐I or CNE2/shRIG‐I

**Figure 8:**
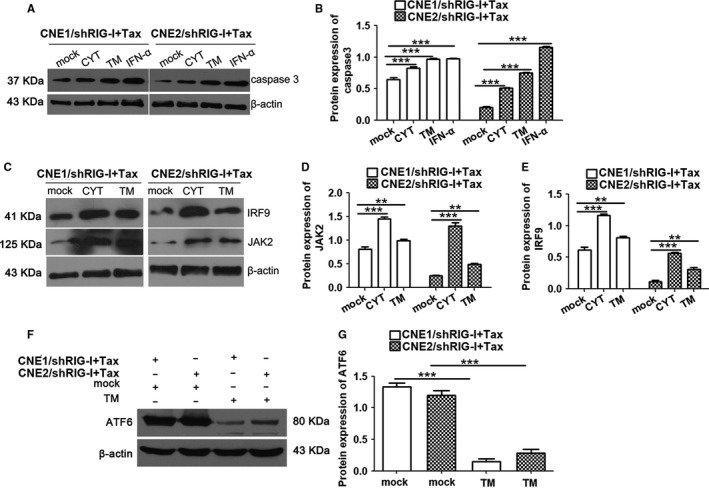
Effects of JAK2, IFN, and endoplasmic reticulum stress response signaling pathways on paclitaxel resistance in nasopharyngeal carcinoma. A and B, CNE1/shRIG‐I and CNE2/shRIG‐I cells were cotreated with CYT, TM, IFN, and paclitaxel (20 μg/mL) for 24 h. Western blotting was used to quantify caspase‐3 expression. C‐E, CNE1/shRIG‐I and CNE2/shRIG‐I cells were cotreated with CYT, TM, and paclitaxel (20 μg/mL) for 24 h. Western blotting was used to quantify JAK2 and IRF9 expression. F and G, CNE1/shRIG‐I and CNE2/shRIG‐I cells were cotreated with CYT, TM, and paclitaxel (20 μg/mL) for 24 h. Western blotting was used to quantify XBP1 and ATF6 expression. Data represent the mean ± SEM (n = 3), **P < .01, ***P < .001 vs CNE1/shRIG‐I or CNE2/shRIG‐I

To verify our hypothesis, we treated CNE1/shRIG‐I and CNE2/shRIG‐I cells with JAK activators (Diosgenin, CYT), ER stress activator (tunicamycin, TM), and IFN (IFN‐a protein) signaling pathway activators. Apoptotic changes were evaluated using MTT assays and flow cytometry. Western blotting was used to measure changes in caspase‐3 expression. When CYT, TM, IFN‐α, and paclitaxel were used together to treat CNE1/shRIG‐I and CNE2/shRIG‐I cells, the number of apoptotic cells was increased in the CYT, TM, and IFN‐α treatment group compared with the control group (IC50: CNE1:CYT/TM/IFN‐a vs mock: 51.55 μg/mL, 63.87 μg/mL, 57.54 μg/mL vs 89.75 μg/mL; CNE2:CYT/TM/IFN‐a vs mock: 64.24 μg/mL, 50.68 μg/mL, 47.62 μg/mL vs 84.64 μg/mL, respectively). This finding reveals that activation of the JAK, ER stress response, and IFN signaling pathways promotes paclitaxel sensitivity in CNE1/shRIG‐I cells and cellular apoptosis (Figure 7A‐D). Similar results were obtained for CNE2/shRIG‐I cells (Figure 8B‐E). Cell cycle experiments revealed that cotreating CNE1/shRIG‐I and CNE2/shRIG‐I cells with CYT, TM, IFN‐α, and paclitaxel produced a G1 block, which subsequently inhibited cell proliferation (Figure 7F‐H).

Western blotting revealed that caspase‐3 expression was increased in the CYT, TM, IFN‐α, and paclitaxel cotreatment group compared with that in the control group (Figure 8A,B). Thus, activation of the JAK, ER stress, and IFN signaling pathways during paclitaxel treatment significantly promoted cellular apoptosis.

Similarly, we measured changes in protein expression after cotreatment with pathway activators (IFN‐a, TM, and CYT) and paclitaxel. The results were similar to those from our previous studies. After CYT or TM treatment, JAK2 and IRF9 (JAK signaling pathway) expression increased (Figure 8C‐E). After TM treatment, the expression of ER stress‐associated factors ATF6 was also increased (Figure 8F,G). These data suggest mutual regulatory links between the ER stress response and JAK/IFN signaling pathways. In summary, we demonstrated that RIG‐I regulates paclitaxel resistance in NPC cells by regulating the JAK2/IFN and ER stress response signaling pathways.

## 4 DISCUSSION

Chemoradiotherapy resistance is a major obstacle in the treatment of numerous cancers.^27^ Poor prognosis and high mortality rates are intimately associated with paclitaxel resistance in advanced NPC patients.^28^ Therefore, studying chemoradiotherapy resistance mechanisms in NPC is important for improving NPC treatments. RIG‐I is a pattern‐recognition receptor on the cell membrane that has been proposed as a target for psoriasis treatment.^29^ In addition, coencapsulation of RIG‐I‐specific ligands and antigenic peptides decreases the growth rate of cells in patients with colorectal cancer and can prevent liver metastases.^29^ PUVA has been shown to activate RIG‐I and promote apoptosis in skin cancer cells.^30^ However, the role of RIG‐I in NPC has not been reported. In the current study, we examined the function of RIG‐I in chemoradiotherapy resistance in NPC and sought to identify the underlying mechanisms.

RIG‐I expression was reduced in radiotherapy‐ and paclitaxel‐resistant NPC cells. In CNE1 and CNE2 NPC cell lines, RIG‐I expression increased with increasing paclitaxel concentration or radiation dosage. RIG‐I overexpression in radiotherapy‐resistant NPC cells induced cellular apoptosis. Experiments in nude mice revealed that the growth of RIG‐I‐transfected CNE1‐CR tumors was inhibited. All of these results demonstrated that RIG‐I increases the sensitivity of NPC cells to radiotherapy and chemotherapy. Similar results were obtained for paclitaxel‐resistant NPC cells: in paclitaxel‐resistant CNE‐1 cells, RIG‐I knockdown led to a decrease in cellular apoptosis and a block at the G1/S cell cycle transition. These results are consistent with previous studies.

To identify the molecular mechanisms that underlie the RIG‐I‐mediated regulation of paclitaxel resistance, we used microarrays to screen for differentially expressed genes. JAK, IFN, and ER stress response signaling pathway factors exhibited reduced expression in the RIG‐I knockdown group, together with the increased expression of relevant signaling pathway components. These finding suggest that RIG‐I regulation of paclitaxel resistance may be associated with the JAK/IFN and ER stress response signaling pathways. Increasing evidence suggests that JAK signaling is associated with drug resistance in tumor cells.^31^, ^32^ For example, IFNα/β activation can induce STAT1/2 and the IFN regulatory factor IRF9 in the nucleus via JAK signaling. These factors form a complex to activate the P53 promoter,^33^, ^34^ which is an important observation because P53‐dependent apoptosis is the major route by which DNA damaging chemotherapy drugs induce cellular apoptosis.^33^ The combined use of JAK2 and HSP90 inhibitors resulted in improved efficacy in drug‐resistant chronic leukemia,^35^ and inhibiting JAK2 activity can reverse paclitaxel resistance in human ovarian cancer cells.^36^ Our results are consistent with these reports. In the present study, drug‐resistant exhibited reduced RIG‐I expression after paclitaxel treatment. Paclitaxel sensitivity in drug‐resistant NPC cells, which express low levels of RIG‐I, was reversed after treatment with JAK/IFN pathway activators. Bax and caspase‐3 expression was also induced, suggesting that cellular apoptosis was induced. These findings demonstrate that RIG‐I regulates paclitaxel resistance in NPC cells by regulating the JAK2/IFN signaling pathway.

The ER is a unique organelle and plays a key role in biosynthesis.^37^, ^38^ Similarly, ER stress‐induced autophagy and apoptosis regulate chemotherapy resistance in tumor cells.^39^ In addition, ER stress inhibits proliferation and drug resistance in multiple myeloma via the PI3K/Akt/mTOR signaling pathway.^40^, ^41^, ^42^, ^43^ ER stress can also regulate CRT/ER p57 complex expression to affect doxorubicin resistance in endometrial cancer.^44^ Cisplatin induces apoptosis in triple‐negative breast cancer cells through ER stress and the calpain1 pathway.^39^ In our study, RIG‐I knockdown in paclitaxel‐resistant NPC cells caused reduced expression of the apoptosis‐associated factors Bax and caspase‐3. In addition, the expression of ER stress response markers was decreased, consistent with previous reports. We also found that treating paclitaxel‐resistant NPC cells with ER stress inducers alleviated paclitaxel resistance. Thus, RIG‐I regulates chemotherapy resistance in paclitaxel‐resistant NPC cells through ER stress. ER stress also regulates JAK signaling to affect tumor progression, such as the inhibition of STAT3‐mediated liver gluconeogenesis via dephosphorylation and deacetylation.^45^ ER stress regulates GH‐induced JAK2 signal transduction and the activation of STAT5 signaling.^46^ In the present study, activation of the ER stress signaling pathway led to increased expression of the JAK2 signaling pathway factors JAK2 and IRF9 was increased. Thus, RIG‐I mediates cellular apoptosis by activating the JAK/IFN and ER stress pathways, thereby affecting paclitaxel resistance in NPC cells.

## 5 CONCLUSIONS

RIG‐I promotes IFN/JAK2 and ER stress response‐mediated apoptosis to inhibit chemoradiation resistance in nasopharyngeal carcinoma.

## CONFLICT OF INTEREST

The authors declare that they have no conflict of interest.
