# Supplementary figures and images for: RIG‐I promotes IFN/JAK2 expression and the endoplasmic reticulum stress response to inhibit chemoradiation resistance in nasopharyngeal carcinoma
Source: Cancer Med. 2019 Aug 28;8(14):6344–57. doi: 10.1002/cam4.2501 (PMC6797570; doi:10.1002/cam4.2501)

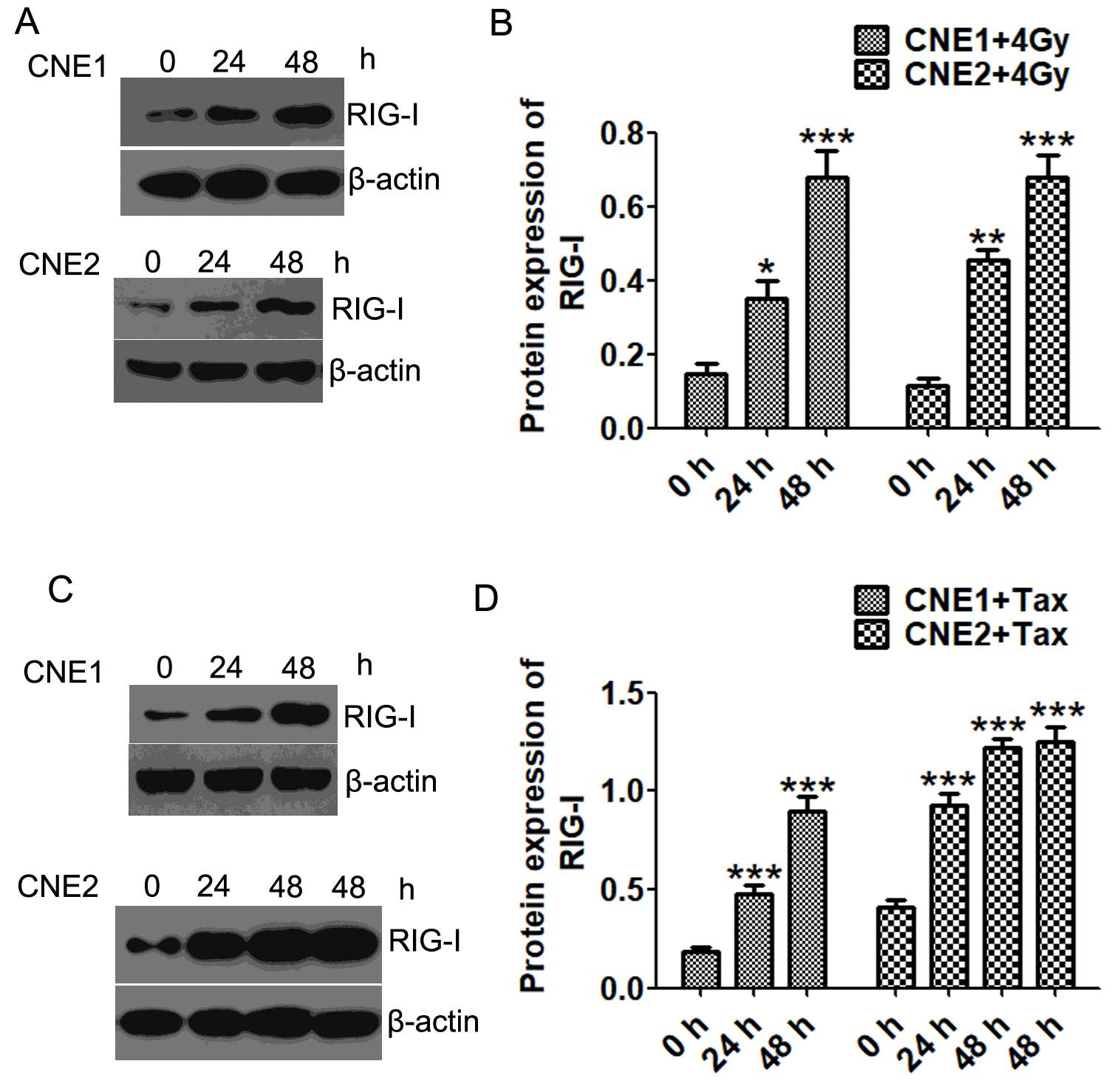

Supplement: Supplementary file 1 [file CAM4-8-6344-s001.tif]

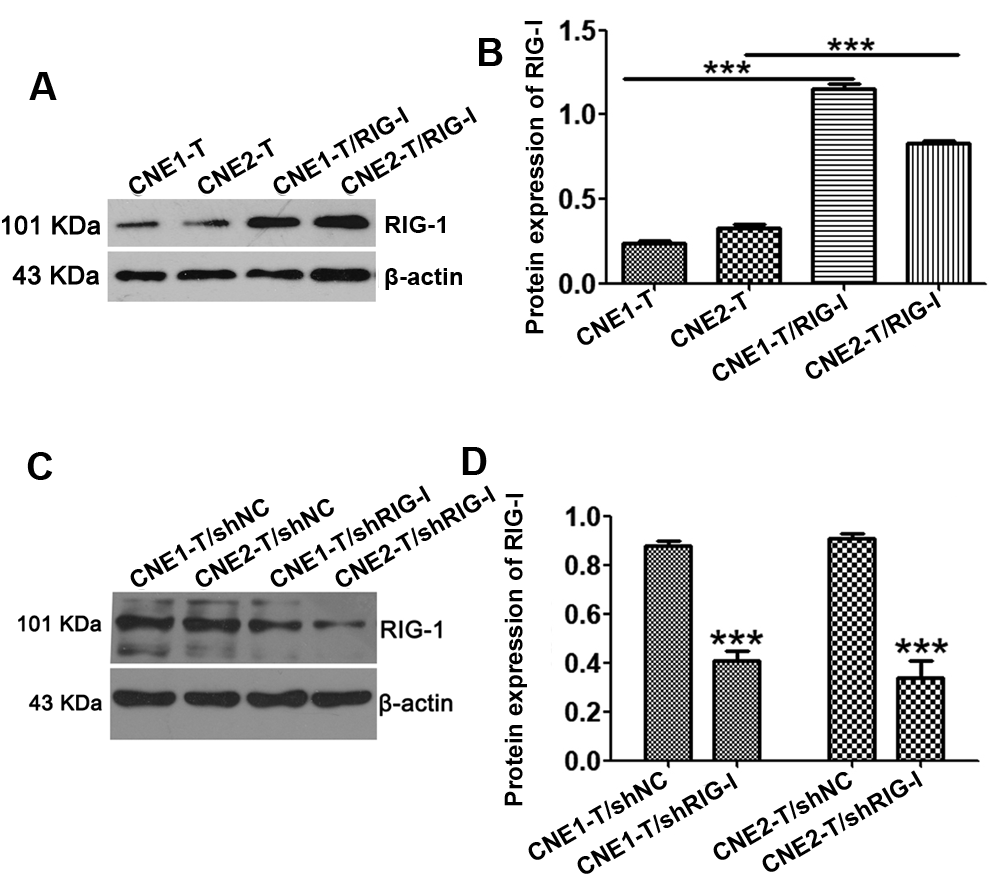

Supplement: Supplementary file 2 [file CAM4-8-6344-s002.tif]
